# Nitric oxide stimulates early egress of *Toxoplasma gondii* tachyzoites from Human foreskin fibroblast cells

**DOI:** 10.1186/s13071-015-1037-5

**Published:** 2015-08-13

**Authors:** Xinlei Yan, Yongsheng Ji, Xianyong Liu, Xun Suo

**Affiliations:** State Key Laboratory of Agrobiotechnology, China Agricultural University, Beijing, 100193 China; National Animal Protozoa Laboratory & College of Veterinary Medicine, China Agricultural University, Beijing, 100193 China; Key Laboratory of Zoonosis of Ministry of Agriculture & College of Veterinary Medicine, China Agricultural University, Beijing, 100193 China

**Keywords:** *Toxoplasma gondii*, *Nitric oxide*, *Non-immune cells*

## Abstract

**Background:**

Egress is a vital step in the life cycle of *Toxoplasma gondii* which attracts attentions of many groups. Previous studies have shown that exogenous nitric oxide (NO) stimulates the early egress of *T. gondii* from infected peritoneal macrophages, a kind of immune cells. However, because *Toxoplasma* forms cysts in brain and muscle tissues, the development of autonomous immunity in non-immune cells is vital for limiting parasite burden and cyst formation. Therefore, we attempted to investigate whether exogenous NO could induce the early egress of *T. gondii* from infected non-immune cells.

**Methods:**

*T. gondii* tachyzoites were cultured in human foreskin fibroblast (HFF) cells and were then treated with NO released by sodium nitroferricyanide (III) dihydrate (SNP). The egressed parasites were analysed by flow cytometry.

**Results:**

The results showed that NO induced the early egress of parasites from HFF cells before completing their intracellular life cycles. We also found that the occurrence of egress was dependent on intracellular calcium (Ca^2+^) levels and the mobility of the parasite. Compared with freshly isolated tachyzoites, the developmental ability and virulence of egressed tachyzoites presented no difference.

**Conclusions:**

Taken together, our findings demonstrate a novel assay for the analysis of egress signalling mechanisms and an avenue of parasite clearance by hosts of *T. gondii.*

## Background

*Toxoplasma gondii* is an obligate intracellular apicomplexan parasite that infects a wide range of vertebrate hosts including humans [1]. One third of the world’s population has been reported to be chronically infected by *Toxoplasma* [2]. Immuno-compromised individuals, such as those with acquired immunodeficiency syndrome (AIDS), and transplant patients with acute or reactivated infections can develop severe infections, which may even lead to death [3]. A few of the devastating consequences caused by the parasite are due to lysis of the host cell during egress [4]. Egress of *T. gondii* was initially studied by inducing elevated levels of intracellular calcium (Ca^2+^) by ionophore A23187 [5]. Ethanol was also used to produce the secretion of microneme proteins, which results in the early egress of the parasite [6, 7]. Another chemical, dithiothreitol (DTT), causes an acute egress of tachyzoites within 60 sec by activating isoforms of the highly concentrated nucleoside triphosphate hydrolase (NTPase) [8]. In addition, a type of potassium ionophore triggers egress by causing an increase in the cytoplasmic Ca^2+^ levels within the parasite through the inositol-1,4,5-triphosphate (IP_3_) pathway [9].

One of the characterised mechanisms of resistance to *T. gondii* in human non-immune cells involves a disruption of the intracellular life cycle of the parasite. Recently, many studies have focused on early egress from non-immune cells induced by immune molecules. Death receptor ligation in *T. gondii* infected cells results in the early egress of infectious parasites via an active process mediated by the release of intracellular Ca^2+^ [10]. In addition, interferon-γ (IFN-γ)-induced cell death leads to early egress of *Toxoplasma*, which may promote the clearance of the parasite by immune cells [11].

Nitric oxide (NO) is produced by a number of different cells in response to cytokine stimulation and has been found to play roles in immunologically mediated protection against a growing list of protozoans, including *T. gondii* [12]. A previous study indicated that NO production during an acute infection with *T. gondii* can kill intracellular parasites [13], and the opposing effects of NO on the parasite contributed towards the establishment of a chronic state of host parasite equilibrium [14]. Moreover, when the parasites replicate in microlia, their multiplication could be prevented by activating the cells with IFN-γ or lipopolysaccharide (LPS), which is a treatment that upregulates upregulate NO synthase activity [15].

Our recent study uncovered another effect of NO against *T. gondii*: exogenous NO induced the the early exit of tachyzoites from infected macrophages [16]. This finding introduced another type of immune-mediated egress for *Toxoplasma*, which may become a new means for the study of parasite clearance by the immune system of host cells. However, the previous study did not determine whether NO could induce egress of tachyzoites from non-immune cells and exposed little information on the mechanism of this immune-mediated egress. In this study, we attempted to determine whether NO could induce the egress of *T. gondii* tachyzoites from non-immune cells and investigated the mechanism of NO-induced egress. Our results showed that NO could trigger the early egress of *T. gondii* tachyzoites from infected human foreskin fibroblast (HFF) cells by elevating the concentration of the cytoplasmic Ca^2+^ of the parasites and that the occurrence of egress required the parasite motility. Moreover, virulence of the egressed tachyzoites was not decreased. Taken together, our discovery presents a novel assay for the analysis of the signalling mechanisms of egress and the study of parasite clearance by hosts of *T. gondii.*

## Methods

### Parasites and cell culture

Two *T. gondii* strains were used: strain RH and a transgenic strain, RH-YFP, which stably expressed yellow fluorescent protein (YFP) [16]. The strains were grown as tachyzoites in monolayer cultures of HFF cells in Dulbecco’s modified Eagle’s medium (DMEM) supplemented with 10 % foetal bovine serum (FBS). The cultures were maintained at 37 °C in a 5 % CO_2_ atmosphere. Parasite cultures were carried out in 25-cm^2^ tissue culture flasks, and egress assays were conducted in 24-well tissue culture plates.

### Animals and ethical approval

C57BL/6 mice (6–8-weeks old) were maintained in a pathogen-free facility*.* All animal research was approved by the Beijing Association for Science and Technology (approval ID SYXK (Beijing) 2007–0023) and complied with the guidelines of the Beijing Laboratory Animal Welfare and Ethics of the Beijing Administration Committee of Laboratory Animals.

### SNP-induced egress assay

RH-YFP parasites (2 × 10^5^) were allowed to infect HFF cells for 2 h. Free parasites were washed with PBS three times. After 36 h of growth at 37 °C, the infected HFF cells were then exposed to either different concentrations of sodium nitroferricyanide (III) dihydrate (Sigma) or DMEM carrier for various durations. After treatments, the numbers of free tachyzoites were counted by flow cytometry (C6, Accuri Cytometers, Inc.).

### Video microscopy

The HFF cells were treated with 40 mM SNP 36 h after infection with RH-YFP strain tachyzoites (2 × 10^5^). The progress of egress was observed under a light microscope and video was recorded.

### Inhibition assays

For inhibition assays, two calcium inhibitors, BAPTA, an extracellular Ca^2+^ chelator and BAPTA-AM, an intracellular Ca^2+^ chelator, were utilised. HFF cells were infected with RH-YFP parasites (2 × 10^5^) for 36 h. Cell cultures were then pre-incubated with 10 μM BAPTA or BAPTA-AM for 30 min. After the pre-incubation, 40 mM SNP was added to the cultures for 30 min, and the numbers of egressed parasites were determined by flow cytometry. To test the effect of disrupting actin-dependent parasite motility on NO-induced egress, intracellular tachyzoites were treated for 30 min with 10 μM cytochalasin D (Sigma), then co-cultured with 40 mM SNP for 30 min. The efficiency of egress was then determined as described above.

### Virulence assay

RH-YFP tachyzoites were allowed to grow in HFF cells for 36 h in 25-cm^2^ tissue culture flasks and were then treated with 40 mM SNP for 30 min. The cell culture medium containing the egressed parasites was centrifuged at 1500 rpm for 10 min. The number of tachyzoites was counted using a blood cell counting plate. For *in vitro* assays, 2 × 10^5^ egressed tachyzoites were added to HFF cells for 24 h, and the number of parasites per vacuole was determined by microscopic examination [17]. For *in vivo* assay, the egressed tachyzoites were used to infect C57BL/6 mice intraperitoneally, and the survival rate after acute challenge was confirmed.

### Statistical analysis

All statistical analyses were processed by the SPSS15.0 Data Editor software (SPSS Inc., Chicago, IL), and all data are expressed as means ± SD values.

## Results

### SNP-released NO triggered early egress of *T. gondii* tachyzoites from infected HFF cells

**Fig. 1:**
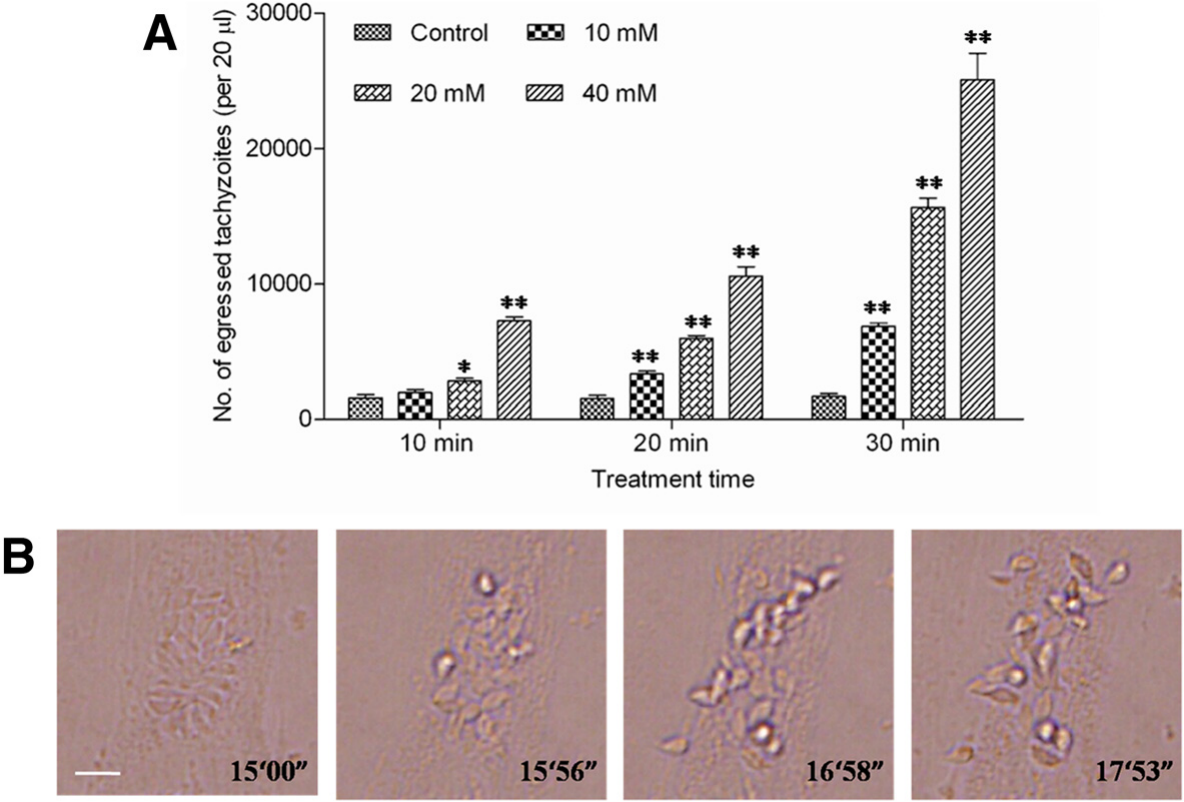
NO released by SNP induced egress of *T. gondii* from infected HFFs. **a** HFFs were infected with *T. gondii* for approximately 36 h, followed by treatment with different concentrations of SNP for the given time periods. Egressed parasites were suspended in 500 μL of DMEM, and 20 μL suspensions were analysed by flow cytometry. The data in the figure show the means ± SEMs of five replicates and represent three independent experiments. *, *P* < 0.05; **, *P* < 0.01, comparing SNP with DMEM at the respective time point. **b** Observation of the egress of tachyzoites via video record. HFFs were incubated with the parasites for 36 h and then treated with 40 mM SNP for 30 min. Video were captured between 15 min and 18 min after SNP treatment and 4 frames were selected and displayed here. Bar: 10 μm

A previous study reported that exogenous NO released by SNP could trigger the egress of *T. gondii* tachyzoites from infected peritoneal macrophages [16]. To investigate whether NO could induce egress of tachyzoite from infected-HFF cells, we treated *T. gondii* tachyzoite-infected HFFs with different concentrations of SNP for different durations as described in the literature [16]. As shown in Fig. 1a, infected HFF cells incubated with SNP displayed increased parasite egress compared with the control group. When infected HFF cells were incubated with 40 mM SNP for up to 30 min, a maximum number of free parasites was observed. By virtue of the video record from microscopy, we observed a large number of tachyzoites egressed from infected cells during treatment with 40 mM SNP (Fig. 1b).

### NO-induced egress was dependent on calcium signalling of the parasites

**Fig. 2:**
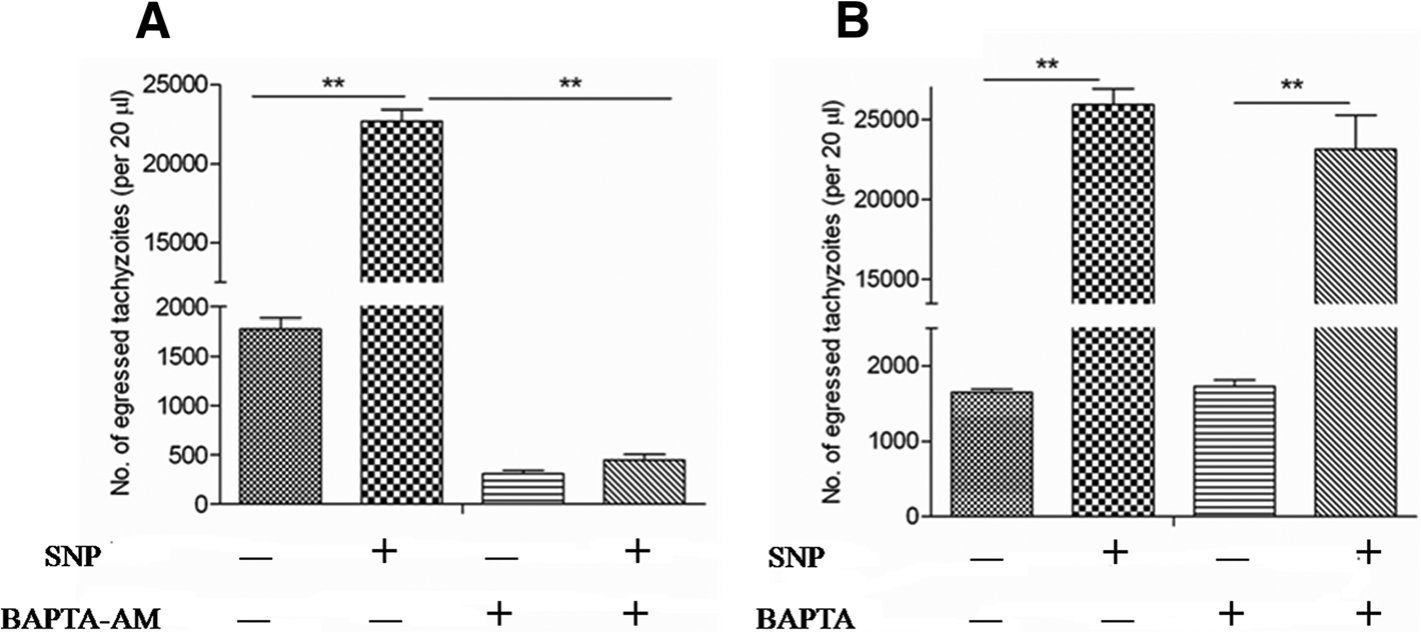
Exogenous NO-induced egress of *T. gondii* from HFFs was dependent on calcium flux of the parasite. Tachyzoite-infected HFFs were pre-treated with BAPTA-AM (**a**) or BAPTA (**b**) to chelate calcium in the parasite cytosol or host cells, respectively, before NO-induced egress assay as described in the Methods section. Egressed parasites were suspended with 500 μL of DMEM, and 20 μL suspensions were analysed by flow cytometry. The data in the figure show the means ± SEMs of four replicates and represent three independent experiments, **P < 0.01

Calcium released from intracellular pools of the parasites was necessary and sufficient to induce many critical events for egress, as was confirmed by many studies [18]. To test whether an increase of parasite Ca^2+^ levels was required during NO-induced egress, the membrane-permeable calcium chelator BAPTA-AM was co-cultured with infected cells before SNP treatment. It was found that treatment of the parasites with BAPTA-AM completely prevented egress (Fig. 2a). Interestingly, we found that the BAPTA chelation of the extracellular Ca^2+^ of the parasite had no effects on the NO-induced egress (Fig. 2b), which suggested that rather than an increase in the Ca^2+^ flux in the host cell cytoplasm, an increase in the parasite Ca^2+^ levels was critical for *Toxoplasma* egress.

### Parasite motility was essential for NO-induced egress

**Fig. 3:**
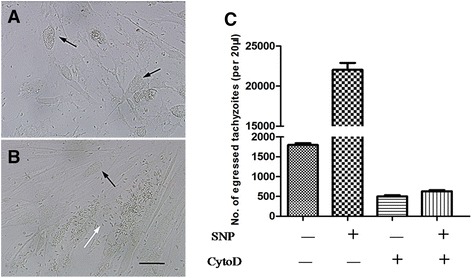
Impairment of parasitic motility impeded NO-induced egress of *T. gondii* from HFFs. NO-induced egress after pre-treatment with cytochalasin D (**a**) or DMEM (**b**) was observed by microscopy. Intact parasitophorous vacuole (black arrow), lytic parasitophorous vacuole (white arrow). Bar: 20 μm. **c** Tachyzoite- infected HFFs were pre-treated with cytochalasin D before NO-induced egress assay. Egressed parasites were suspended with 500 μL of DMEM, and 20 μL suspensions were analysed by flow cytometry. The data in the figure show the means ± SEMs of five replicates and represent three independent experiments, **P < 0.01

Gliding motility is essential for *T. gondii* tachyzoites to traverse across substrates, migrate through tissues, and invade into and finally egress from their host cells [4]. Thus, we hypothesised that the effect of NO on egress should be dependent on the motility machinery of the parasite. To test this hypothesis, we studied the effect of the actin inhibitor cytochalasin D, which can completely block the actin microfilaments and gliding motility of *T. gondii* [19]. When intracellular tachyzoites were treated with NO in the presence of cytochalasin D, the parasites lost their ability to egress from HFF cells (Figs. 3a, b, and c). This result suggested that gliding motility is a crucial step in NO-induced egress.

### Occurrence of NO-induced egress had no effects on virulence of egressed parasites

**Fig. 4:**
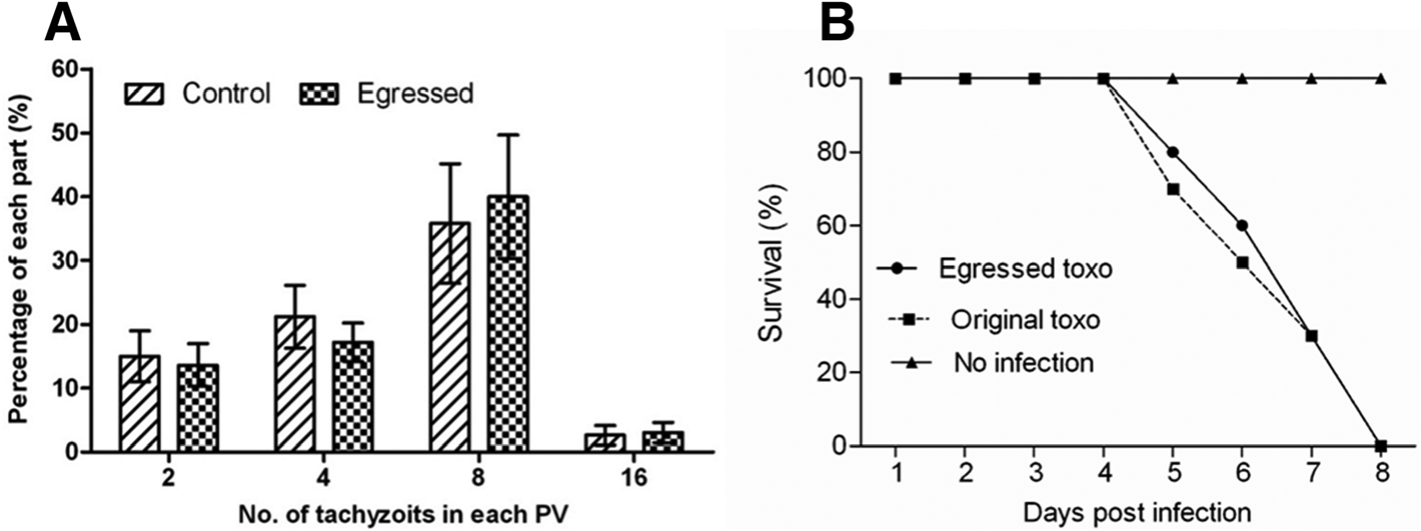
Egressed parasites had similar *in vitro* growth and virulence as control parasites. **a** HFFs were infected with egressed *T. gondii* induced by NO or naturally released parasites. Twenty-four hours later, the cells were fixed and the number of parasites per vacuole in eight fields was counted and expressed as a percentage of the total number of parasites in the field. The data in the figure show the means ± SDs of five replicates and represent three independent experiments. **b** C57BL/6 mice were *i.p* infected with 10^3^ parasites and the survival rate was recorded every day

Previous studies on the egress of *T. gondii* reported that death receptor ligation could induce parasite egress. However, the egressed tachyzoites would then rapidly infect surrounding cells, including Ag-specific effector cells, which could potentially contribute to a local dissemination of the infection [10]. To investigate whether the parasites were still infectious after NO-induced egress, we collected egressed tachyzoites to infect newly prepared HFF cells or to intraperitoneally inoculate C57BL/6 mice. It was found that both the developmental ability in host cells (Fig. 4a) and the lethal infection in laboratory mice (Fig. 4b) by parasites produced via NO-induced egress were not different from those of normal parasites.

## Discussion

Our results demonstrated that NO released by SNP was able to trigger early egress of *T. gondii* tachyzoites from non-immune cells *in vitro*. Moreover, these processes were revealed to be Ca^2+^-dependent as inhibition of parasite intracellular Ca^2+^ prevented this phenomenon. Our further experiments showed that the occurrence of egress required parasite motility, and the egressed tachyzoites lost no virulence. Taken together, our study investigated the mechanism of NO-induced tachyzoite egress and presented a new mechanism of parasite clearance.

*T. gondii* establishes a life-long infection in hosts by forming cysts in brain and muscle tissues. Therefore, cell-autonomous immunity in non-immune cells is crucial for limiting parasite burden and cyst formation [11]. Previous studies have demonstrated that death receptor ligation and IFN-γ induce the egress of *T. gondii* tachyzoites from non-immune cells *in vitro* [10, 11]. Here, we found that NO is another immune molecule that can induce the early egress of *T. gondii* tachyzoites from non-immune cells. Moreover, we found that the the egressed tachyzoites lost no virulence, but NO stimulated the egress of parasites from HFF cells 36 h after infection, long before natural egress occurred, and thereby disturbed the proliferation of the parasite. If this kind of immune-mediated egress existed *in vivo*, it may limit replication of the tachyzoites in non-immune cells and lower the parasite burden of the host. Moreover, the occurrence of egress before complete proliferation shortens the time of the parasite in parasitophorous vacuole which promotes the clearance of the parasites by immune cells of the hosts, like macrophages.

The study of the egress of *T. gondii* has attracted attention from many research groups for nearly four decades. *T. gondii* egress is a crucial step in the life cycle of the parasite because it allows parasites to shuttle into neighbouring cells for further development. Many studies have shown that intracellular Ca^2+^ levels of the parasite play important roles in regulating the egress of parasites from host cells in response to a variety of extracellular signals [20–22]. Our study found that rather than an increase in the Ca^2+^ levels in the host cell, an increase of Ca^2+^ levels in the parasite was critical for the NO-induced egress of *T. gondii* tachyzoites. Moreover, in *Plasmodium falciparum*, an increase in the intracellular Ca^2+^ levels was found to stimulate parasite egress from infected erythrocytes [23]. Another study showed that the addition of Ca^2+^ ionophores induces the rapid egress of *Neospora caninum* tachyzoites from bovine endothelial cells [24]. Recent research on *Eimeria tenella* revealed that ethanol, a reagent that is able to elevate intracellular Ca^2+^ levels, induces sporozoite egress from primary chicken kidney cells [25]. Consistent with the findings on *T. gondii,* we hypothesised that Ca^2+^ is a universal signalling molecule of the egress process in apicomplexan parasites.

## Conclusions

In conclusion, our research demonstrated that NO is an immune molecule that could induce the early egress of *T. gondii* tachyzoites from non-immune cells and investigated the mechanism of this immune-mediated egress. Future studies will address whether these immune molecules could induce parasite egress from feline intestinal epithelial cells *in vitro* and whether the induction of early parasite egress from non-immune cells by immune molecules could occur *in vivo*.
